# Limited Open Reduction and Transepiphyseal Intramedullary Kirschner Wire Fixation for Treatment of Irreducible Distal Radius Diaphyseal Metaphyseal Junction Fracture in Older Children

**DOI:** 10.3389/fped.2022.871044

**Published:** 2022-04-13

**Authors:** Rufa Wang, Liwei Wu, Yinming Wang, Minjie Fan, Yiwei Wang, Bo Ning, Pengfei Zheng

**Affiliations:** ^1^Department of Orthopaedic Surgery, Children’s Hospital of Nanjing Medical University, Nanjing, China; ^2^Department of Pediatric, Children’s Hospital of Nanjing Medical University, Nanjing, China; ^3^National Children’s Medical Center, Children’s Hospital of Fudan University, Shanghai, China

**Keywords:** children, distal radius fracture (DRF), junction, Kirschner wire (K-wire), plate

## Abstract

**Objective:**

This study aimed to compare limited open reduction and transepiphyseal intramedullary fixation with Kirschner wire (LOR-TIKW) versus open reduction and internal fixation with plate and screw (ORIF-PS) for treatment of irreducible distal radius diaphyseal–metaphyseal junction (DMJ) fracture in older children.

**Methods:**

Data of children (aged 10–14 years) treated in our hospital for distal radius DMJ fractures with LOR-TIKW or ORIF-PS from January 2018 to December 2019 were retrospectively analyzed. Follow-up was until radiographic union. Demographic, clinical, and radiographic data; treatment cost; healing time; functional outcome (by Price criteria); complications; and postoperative angulation and displacement were compared between children treated by the two methods. Statistical analysis was performed with alpha set at *P* < 0.05.

**Results:**

A total of 26 children were included: 14 treated with LOR-TIKW and 12 with ORIF-PS. Operation time was less (22.1 min vs. 46.7 min, *P* < 0.0001), surgical incision smaller (2.43 cm vs. 5.00 cm, *P* < 0.0001), cost of internal fixation lower (US$, 40.6 vs. 2020, *P* < 0.0001), and healing time shorter (4.79 weeks vs. 5.64 weeks, *P* = 0.03) with LOR-TIKW; however, postoperative fracture angulation was slightly larger (1.07° vs. 0.83°, *P* = 0.85) and displacement slightly more (0.86 mm vs. 0.58 mm, *P* = 0.44) in the LOR-TIKW group. Rate of union, functional outcome, and complications were not significantly different between the groups.

**Conclusion:**

For irreducible DMJ fracture of distal radius in older children, LOR-TIKW appears to be a promising method with several advantages over ORIF-PS.

## Introduction

Distal radius fracture is the most common fracture in children (1–4). The AO classification system does separately define fracture of the diaphyseal–metaphyseal junction of the distal radius in children (5). In 2010, Lieber et al. (2) proposed the term diametaphyseal transition (DMT) fracture for this injury. Since then, it has come to be known as diaphyseal–metaphyseal junction (DMJ) fracture (6–8). Manual reduction and plaster fixation is the usual treatment for distal radius fractures in children but it is not very effective for DMJ fracture. Displacement is common even after successful reduction because of the small contact area between the two ends of the fracture. Currently, DMJ fracture is treated with open reduction and plate–screw fixation (3, 9, 10) or elastic intramedullary nailing fixation (2, 3, 6–8, 11) or Kirschner wire fixation (2–4, 10), or external fixation (3, 12), the choice depending upon the age of the child, the type of fracture, and the preference of the surgeon. For older children (>10 years old), open reduction and internal fixation with plate and screw (ORIF-PS) is the usual choice.

The ideal treatment method would be minimally invasive, rapid, simple, and economical, while ensuring good outcomes, but none of the above mentioned methods meet all these criteria. We have recently developed a different method—limited open reduction and transepiphyseal intramedullary fixation with Kirschner wire (LOR-TIKW)—for treatment of this fracture. The purpose of this study was to describe our technique and to retrospectively compare its efficacy with that of ORIF-PS in the treatment of distal radius DMJ fracture in children over 10 years old.

## Materials and Methods

The data of children treated in our hospital for distal radius DMJ fractures with LOR-TIKW or ORIF-PS between January 2018 and December 2019 were retrospectively analyzed. The inclusion criteria were (1) child aged 10–14 years; (2) DMJ fracture of distal radius; (3) complete displacement of fracture end or angulation of fracture end exceeding 20°; (4) failure of closed reduction or inability to maintain stable reduction; (5) follow-up until radiographic union was achieved. The exclusion criteria were (1) pathological fracture; (2) presence of other fractures (except distal ulna fracture); (3) other vascular–neural complications; (4) incomplete follow-up data.

The selected patients were separated into two groups according to the surgery performed – ORIF-PS or LOR-TIKW—and demographic, clinical, intraoperative, and postoperative characteristics were compared between the groups.

This study was approved by the Ethics Committee of our hospital. Informed consent was obtained from the parents/guardians before surgery.

### Surgery Method

The surgeries were performed by five different senior pediatric orthopedic surgeons. The choice of LOR-TIKW or ORIF-PS depended on the experience and personal preference of the operating surgeon.

**FIGURE 1:**
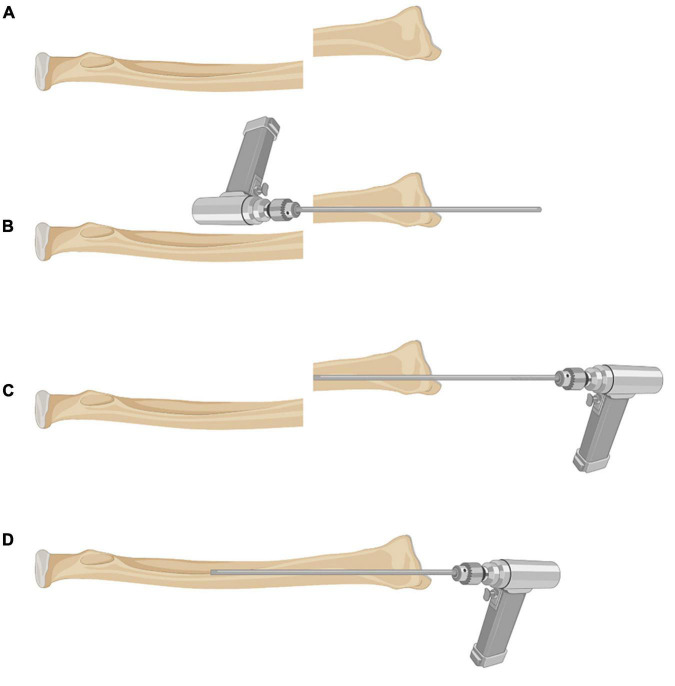
Schematic diagram of surgical technique for LOR-TIKW. **(A)** Distal radius DMJ fracture; **(B)** a smooth 2.0-mm-diameter Kirschner wire was inserted antegradely from the fracture end into the medullary cavity of distal radius, the wire being kept parallel to medullary cavity of distal fragment; **(C)** the distal end of Kirschner wire was retracted into the fracture end; **(D)** the fracture was reduced under direct vision, and the Kirschner wire was retrogradely inserted into the medullary cavity of the proximal radius.

For LOR-TIKW (Figure 1), a dorsal longitudinal incision, approximately 2 cm long, was made centered on the fracture end of the radius to expose the fracture end. A smooth 2.0-mm-diameter Kirschner wire was then inserted antegradely from the fracture end into the medullary cavity of the distal radius, the wire being kept perpendicular to the radial growth plate as far as possible. The Kirschner wire was then punched out through the skin, and the distal end of Kirschner wire was retracted into the fracture end. The fracture was reduced under direct vision, and the Kirschner wire was then retrogradely inserted into the medullary cavity of the proximal radius. C-arm fluoroscopy was used to confirm fracture reduction and correct internal fixation. The end of the Kirschner wire outside the skin was bent 90° and left outside. The incision was closed, and plaster external fixation was applied.

For ORIF-PS, a dorsal or lateral longitudinal incision, approximately 5 cm long, was made centered on the fracture end. The fracture was reduced under direct vision and fixed by plate (straight or T-shaped) and screws. C-arm fluoroscopy was used to confirm fracture reduction and correct internal fixation. The incision was closed, and plaster external fixation was applied.

Distal ulnar fracture, if present, was either left alone or treated with anterograde elastic intramedullary nail fixation, depending on the degree of fracture displacement.

### Data Collection

Demographic and clinical data of the patients were collected from the case records. Radiographs were reviewed, and the fractures were classified as oblique or transverse. Presence of distal ulnar fracture was recorded. Operative time, incision length, and cost of internal fixation were recorded. Postoperative angulation and displacement of fracture in coronal or sagittal planes was noted.

The children were followed up in the outpatient clinic until radiographic union (i.e., callus on three of the four cortical surfaces). The union time was recorded. The plaster was removed after fracture union was confirmed by radiography. In the LOR-TIKW group, the Kirschner wire was removed at the same time. In both groups, functional exercises were started after plaster removal. At final follow-up, functional outcome was graded using the system proposed by Price et al. (13) as “excellent” (able to perform strenuous physical activity and/or loss of forearm rotation ≤ 10°); “good” (mild difficulty in performing strenuous physical activity and/or loss of forearm rotation 11°–30°); “fair” (subjective complaints in performance of daily activities and/or loss of forearm rotation 31°–90°); or “poor” (all other outcomes). Wrist joint function of the affected side was compared with that of the healthy side. Complications were recorded as major or minor. Major complications included need for additional surgery (other than conventional internal fixation removal), non-union, delayed union, re-fracture, malunion, and epiphyseal damage (14). Minor complications included infection of surgical incision and Kirschner wire tail slippage.

### Statistical Methods

GraphPad Prism 6 (GraphPad Software, La Jolla, CA, United states) was used for statistical analysis. Baseline, treatment, and outcome data were compared between the LOR-TIKW group and ORIF-PS group. Continuous variables were summarized as the means (±standard deviation) and compared between groups using the *t*-test. Categorical variables were summarized as percentages and compared using the Fisher exact test. Statistical significance was at *P* < 0.05.

## Results

A total of 26 children (25 boys and 1 girl; mean age, 12.02 ± 1.28 years) were included in this study. While 14 patients received LOR-TIKW, 12 patients received ORIF-PS. Table 1 presents a comparison of characteristics of the two groups. Age, sex, fracture side, fracture type, concurrent ulnar fracture, postoperative deformity (angulation and displacement), follow-up time, healing rate, proportion achieving union, incidence of complications, and functional outcome were not significantly different between the two groups (all *P* > 0.05). However, operation time (22.1 ± 0.69 min vs. 46.7 ± 1.42 min, *P* < 0.0001), incision length (2.43 ± 0.14 cm vs. 5.00 ± 0.25 cm, *P* < 0.0001), implant cost ($40.6 ± 0.0 vs. $2020 ± 27.8, *P* < 0.0001), and time to union (4.79 ± 0.21 weeks vs. 5.64 ± 0.31 weeks, *P* < 0.03) were all significantly lower in the LOR-TIKW group than in the ORIF-PS group (Table 1).

**TABLE 1:** Patient demographic, radiographic data, surgical data, outcome data, and complications.

Parameters	TIKW (*n* = 14)	ORIF (*n* = 12)	*p*
Age (years)	11.8 ± 0.37	12.2 ± 0.34	0.45
Sex (male/female)	14/0	11/1	0.46
Fracture side (L/R)	7/7	9/3	0.25
Fracture pattern			1.0000
Oblique	3	3	
Transverse	11	9	
Ulnar distal fracture	13	11	1.0000
Surgical time (minutes)	22.1 ± 0.69	46.7 ± 1.42	**<0.0001**
Incision length (cm)	2.43 ± 0.14	5.00 ± 0.25	**<0.0001**
Implant cost (US$)	40.6 ± 0.0	2020 ± 27.8	**<0.0001**
Postoperative angular deformity (degrees)	1.07 ± 1.07	0.83 ± 0.56	0.85
Postoperative displacement(mm)	0.86 ± 0.25	0.58 ± 0.23	0.44
Follow-up (months)	13.9 ± 0.41	14.8 ± 0.94	0.36
Achieved union	14	11	0.46
Time to union (weeks)	4.79 ± 0.21	5.64 ± 0.31	**0.03**
Clinical outcome (Price CT)			0.60
Excellent	13	10	
Good	1	1	
Fair	0	0	
Poor	0	1	
Complication			0.39
Non-union	0	0	
Delayed union	0	0	
Refracture	0	1	
Malunion	0	0	
Physeal Arrest	0	0	
Skin Infection	1	1	

*TIKW, limited open reduction and transepiphyseal intramedullary fixation with Kirschner wire; ORIF, open reduction internal fixation with plate and screw; L, Left; R, Right.*

*Values in bold represent a P-value < 0.05.*

**FIGURE 2:**
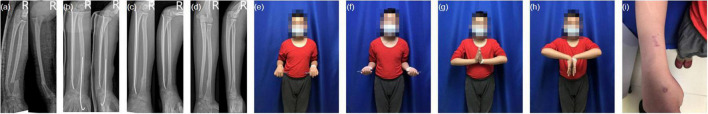
A male patient, 11 years and 11 months old, with fracture of the right radius and ulna. Limited open reduction and transepiphyseal intramedullary fixation with Kirschner wire (2.0 mm diameter) was used to treat the radius fracture and closed antegrade intramedullary nail fixation (2.0 mm diameter) for the ulna fracture. Anteroposterior and lateral radiographs: **(a)** preoperative; **(b)** 1 week postoperative; **(c)** 5 weeks postoperative (removel of Kirschner wire); **(d)** 6 months postoperative (there is no physeal arrest). Final follow-up: **(e)** pronation; **(f)** supination; **(g)** dorsiflexion; **(h)** no limitation of palmer flexion; **(i)** scar.

**FIGURE 3:**
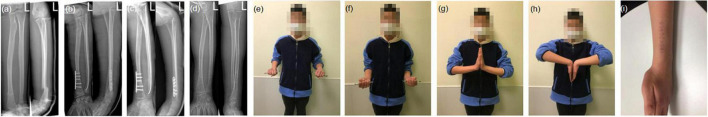
A male patient, 13 years and 3 months old, with fracture of the left radius and ulna. Open reduction internal fixation with one plate and five screws was used to treat the radius fracture and closed antegrade intramedullary nail fixation (2.0 mm diameter) to treat the ulna fracture. Anteroposterior and lateral radiographs: **(a)** preoperative; **(b)** 1 week postoperative; **(c)** 5 weeks postoperative; **(d)** 6 months postoperative. Final follow-up: **(e)** pronation; **(f)** supination; **(g)** dorsiflexion; **(h)** no limitation of palmer flexion; **(i)** scar.

**FIGURE 4:**
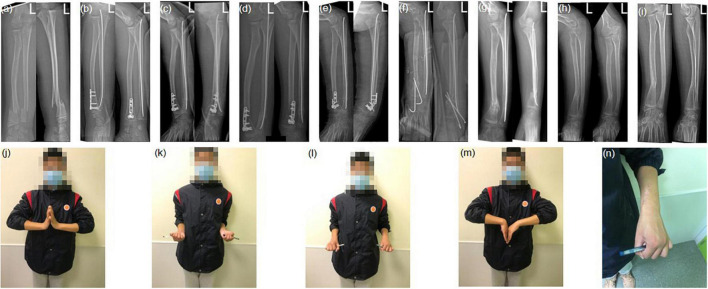
A male patient, 11 years and 9 months old, with fracture of the left radius and ulna. Open reduction internal fixation with one plate and five screws was used to treat the radius fracture and closed antegrade intramedullary nail fixation (2.0 mm diameter) to treat the ulna fracture. Anteroposterior and lateral radiographs: **(a)** preoperative; **(b)** 1 day postoperative; **(c)** 6 weeks postoperative; **(d)** 10 weeks postoperative (delayed union); **(e)** 3 months postoperative (plate fracture); **(f)** 3 months postoperative (reoperation performed with two 2.0-mm-diameter Kirschner wires fixation and iliac crest bone graft); **(g)** 5.5 months postoperative (2.5 months after the reoperation and after removal of the two Kirschner wires); **(h)** 13 months postoperative (after removal of one plate); **(i)** 16 months postoperative (final follow-up). Final follow-up: **(j)** pronation; **(k)** supination; **(l)** no limitation of dorsiflexion; **(m)** there is 20° limitation of palmar flexion; **(n)** scar.

In the LOR-TIKW group, the mean follow-up time was 13.9 ± 0.41 months and all children (100%) achieved union with the mean union time of 4.79 ± 0.21 weeks. Functional exercises were started after removal of the plaster and Kirschner wires. At the final follow-up, Price CT functional evaluation showed excellent in 13 cases and good in 1 case. There were no major complications, but 1/14 patient developed a skin infection 2 weeks after surgery due to irritation by the Kirschner wire tail; the infection healed with regular dressing changes. In ORIF-PS group, the mean follow-up time was 14.8 ± 0.94 months and 11 children (91.7%) achieved union with the mean union time of 5.64 ± 0.31 weeks. Functional exercises were started after removal of the plaster and Kirschner wires. At the final follow-up, Price CT functional evaluation showed excellent in 10 cases, good in 1 case and poor in 1 case. One child (8.33%) had major complication. This child had delayed union at 10 weeks after surgery, followed by the steel plate broke and re-fracture at 3 months after surgery. He was treated with plate removal, iliac crest bone graft, and fixation with two 2-mm-diameter Kirschner wires. Union was achieved at 13 months after the injury. A minor complication occurred in another patient (1/12). This child had infection of the surgical incision at 3 days after surgery; the infection subsided with dressing changes and antibiotic treatment. Internal fixation was removed in all patients. Figures 2–4 showed representative images of patients from the two groups.

## Discussion

The Pediatric Comprehensive Classification of Long Bone Fractures (PCCF) (5) defines the metaphysis as the square area containing the widest part of the double growth plate on the anteroposterior radiograph, but the diaphyseal–metaphyseal junctional area is not defined. Lieber et al. (2) first proposed the term diametaphyseal transition (DMT) zone for the square area containing the widest part of distal ulnar and radial growth plates minus the widest part of the distal radius growth plate on the anteroposterior radiograph. In this study, we used the definition of Lieber et al. to select the study population.

For older children, adequate initial reduction of DMJ fracture is crucial as revision surgery for malunion may have a high risk of complications, and not be successful due to poor plasticity (15–17). Plasticity depends on both the age of the child and the location of fracture: the older the child and the closer the fracture is to the proximal end, the poorer the plasticity. Rodríguez-Merchán et al. (18) and Ploegmakers et al. (19) recommended surgical treatment for children over 10 years with forearm fractures that cannot be reduced manually. Open reduction and internal fixation is advisable if manual reduction fails or there is re-displacement after reduction. Hoël et al. (20) have successfully used the Kirschner wire poking reduction technique, but we believe that the method is associated with high risk of damage to surrounding blood vessels, tendons, and nerves and, moreover, reduction of soft issue embedded in the fracture can be difficult. We therefore prefer open reduction. For distal radius metaphyseal fracture, crossed internal fixation with Kirschner wire is safe and effective (2–4, 10) and, for pediatric radial diaphyseal fracture, elastic intramedullary nail fixation is currently the preferred option (2, 3, 21, 22). However, for distal radius DMJ fracture in children, the fracture location is too distal for intramedullary nail fixation and too proximal for crossed Kirschner wire fixation. We therefore developed a technique of limited open reduction and transepiphyseal intramedullary fixation with Kirschner wire applied for this kind of fracture.

In this study, we compared our technique with conventional ORIF-PS. In terms of postoperative deformity, compared to the ORIF-PS group, the mean postoperative fracture angulation was slightly greater in the LOR-TIKW group, and the mean postoperative fracture displacement was slightly more in the LOR-TIKW group. However, there was no significant difference between the two groups. The anatomical reduction of the fracture was obtained during the operation, while because of the possible micromovement of fracture end, the postoperative fracture angluation was slightly larger and the fracture displacement was slightly more. Finally, the LOR-TIKW group could achieve the same alignment effect as the ORIF-PS group, and there was no difference in treatment. In terms of time of plaster protection, compared with ORIF-PS group, the LOR-TIKW group had a smaller incision, less periosteum stripping and less blood supply destruction of fracture end. Moreover, the micromovement of fracture end in the LOR-TIKW group accelerated fracture union. Yung PS et al. (22) reported that a certain degree of micromovement at the fracture end could stimulate the formation of bridging callus, which may promote fracture union. In terms of re-fracture rate, there was no major complications in the LOR-TIKW group. One case in the ORIF-PS group had major complications, who presented with a broken steel plate and re-fracture at 3 months after surgery. Considering the short follow-up time of both groups, the re-fracture rate may be underestimated, so the patients in both groups continued to be followed up later to record the incidence of re-fracture. Although satisfactory outcome and fracture union could be achieved with both methods, operation time was shorter (by ∼24 min), the surgical incision shorter (by ∼2.6 cm), and the cost of internal fixation lower (by ∼US$1080) with LOR-TIKW. LOR-TIKW has one shortcoming that is a risk of iatrogenic epiphyseal injury. Fortunately, none of 14 children who underwent LOR-TIKW in this study had growth imbalance caused by epiphyseal injury. This may because it was generally accepted that smooth Kirschner wire of 2.0 mm or less in diameter would not affect the epiphysis (21–23). The other reason maybe that Kirschner wires were inserted successfully in one-time, even if only got good alignment of the fracture but still left slightly displaced, it would not affect the later union and function recovery.

In conclusion, for irreducible DMJ fracture of distal radius in older children, LOR-TIKW appears to be a promising method with several advantages over ORIF-PS.

## Data Availability Statement

The raw data supporting the conclusions of this article will be made available by the authors, without undue reservation.

## Ethics Statement

This study was approved by the Ethics Committee of our hospital. Informed consent was obtained from the parents/guardians before surgery.

## Author Contributions

PZ and BN contributed to the conception and design of the study and revised the manuscript. RW, LW, and YMW organized the database and wrote the first draft of the manuscript. MF and YWW performed the statistical analysis. All authors contributed to the manuscript revision, read, and approved the submitted version.

## Conflict of Interest

The authors declare that the research was conducted in the absence of any commercial or financial relationships that could be construed as a potential conflict of interest.

## Publisher’s Note

All claims expressed in this article are solely those of the authors and do not necessarily represent those of their affiliated organizations, or those of the publisher, the editors and the reviewers. Any product that may be evaluated in this article, or claim that may be made by its manufacturer, is not guaranteed or endorsed by the publisher.
